# A Multi-Disciplinary Approach to Remote Sensing through Low-Cost UAVs

**DOI:** 10.3390/s17061411

**Published:** 2017-06-16

**Authors:** Gabriela Calvario, Basilio Sierra, Teresa E. Alarcón, Carmen Hernandez, Oscar Dalmau

**Affiliations:** 1 Departamento de Ciencias de la Computación e Inteligencia Artificial, Universidad del País Vasco UPV/EHU, 20018 Donostia-San Sebastián, Spain; b.sierra@ehu.eus (B.S.); mamen.hernandez@ehu.es (C.H.); 2 Centro Universitario de los Valles, Carretera Guadalajara - Ameca Km. 45.5, CP 46600 Ameca, Jalisco, México; teresa.alarcon@profesores.valles.udg.mx; 3 Centro de Investigación en Matemáticas, Jalisco SN, Col. Valenciana, CP 36240, Guanajuato, México; dalmau@cimat.mx

**Keywords:** UAV, data mining, computer vision, geomatics, agave monitoring

## Abstract

The use of Unmanned Aerial Vehicles (UAVs) based on remote sensing has generated low cost monitoring, since the data can be acquired quickly and easily. This paper reports the experience related to agave crop analysis with a low cost UAV. The data were processed by traditional photogrammetric flow and data extraction techniques were applied to extract new layers and separate the agave plants from weeds and other elements of the environment. Our proposal combines elements of photogrammetry, computer vision, data mining, geomatics and computer science. This fusion leads to very interesting results in agave control. This paper aims to demonstrate the potential of UAV monitoring in agave crops and the importance of information processing with reliable data flow.

## 1. Introduction

Remote sensing (RS) through Unmanned Aerial Vehicles (UAVs), is not only a new topic of research in the civil field, but also an alternative to conventional platforms, for the acquisition of data with infinite possibilities. Some examples of UAVs are: vegetation monitoring [1], forest inspection, mapping of territorial coverage [2], disaster response, construction monitoring [3], industrial and residential inspection, three-dimensional photogrammetric models, hydrocarbon pipeline monitoring and coastal surveillance [4]. Regarding the advantages of UAV, we also can highlight the importance to facilitate activities that have a detrimental effect on humans. Currently, we can remotely execute risk tasks, such as flying over contaminated areas, or places with high levels of radiation or in danger of collapse.

The support provided to agriculture through UAVs can be used to create alternatives with greater versatility and low cost. UAV technology in conjunction with other disciplines and fields of research are generating new applications in agriculture, such as crop identification, monitoring and mapping of cultivated areas, pest detection, crop yield estimation and prediction of anomalies. The check schedule in the field is more adjustable for the user. Therefore, monitoring crops through UAV can be a good tool for decision-making, management and planning of public policies in the agriculture. As the satellite sensors, UAV also allows for obtaining reliable data but in a more economical way.

Another important fact that has a direct impact on agriculture monitoring is climate change. This fact generates the need for low-cost and multitemporal monitoring. The increase of CO${}_{2}$ leads to a remarkable change in the growth and maturation of vegetation [5], which causes problems in the crops. Through a UAV remote sensor, it is possible to detect the location of the weed within an agricultural field, and, consequently, the right herbicide in the correct amount can be applied in a specific area. UAV sensors with high spatial resolution also generate data, which allows for discriminating different types of weeds, crops and textures.

Satellite imagery and aerial photography play an important role in agricultural monitoring; they are a robust research tool for monitoring and evaluating large scale crop inventory; however, there is a limitation: free sources of satellite imagery do not provide images with enough spatial resolution [6] as those given by the UAVs.

Precision farming requires frequent information on crop conditions. UAV is an adequate tool to systematically provide information with a high resolution and low-cost in real time. However, an important fact to be taken into account is that the farmer is often not very familiar with the use of images [6]. Another aspect to consider is that the flight session of the UAVs is limited by the battery charge, which leads to short periods of use and limited areas. In this respect, it is worth mentioning their vulnerability to cyber attacks and being knocked down, and their high risk of mismanagement by the users [7]. Nowadays, there is an increasing use of UAVs in the monitoring area, and the market for ultra-light cameras with spectral and hyperspectral ranges [8,9] has grown. The use of technologies such as special sensors [10,11], synthetic aperture radar and thermal sensors in UAVs is already remarkable. The growth of digital image processing tools is also observed (for example, Skycatch [12], dronedeploy [13], among others). A report about UAV sales estimated about 13.22 billion dollars in 2016 year and speculated an increase of up to 28.27 billion dollars by 2022 [14].

The relationship between computer vision, data mining [15], geomatics, computer science and photogrammetry are generating a very interesting multidisciplinary environment in the extraction of information and its treatment by the means of UAVs [16].

Remote sensing technology allows a feasible solution for crop monitoring. This technology together with computer science leads to monitoring, detecting and classifying different types of crops. In Ref. [17], six different supervised classifiers are implemented with the purpose of monitoring and classifying corn crops through RapidEye images. The studied supervised techniques were: Maximum Likelihood [18,19], Mahalanobis and Minimum distances [20], Spectral Angle Mapper [21,22], the Parallelepiped method [23] and SVM (support vector machine) [24,25,26]. Winner-takes-all (WTA) is the final classifier [27], which is an ensemble based classifier, and, for the research in [17], WTA considers the voting derived from the six mentioned techniques. In [28,29,30], an artificial neural network (ANN) is used to predict vegetation parameters and crop yields using data from satellite images. Vegetation indices are widely used to enhance the vegetation information in satellite images with the purpose of monitoring [31,32]. The mentioned works face the precision problem during the monitoring, detection and classification process of the crops. The problems are not only due to selected classifiers, but also due to the spatial resolution of the satellite sensor, and, even though this parameter is improving constantly because of the new technology, the access to high spatial resolution data also is high-cost. Therefore, achieving a high resolution is a problem for satellite images users in the agriculture. On the contrary, through UAVs, it is possible to achieve high spatial resolution at a low-cost in comparison with satellite sensors.

Agriculture is a very important item for the economy of any country. In Mexico, agriculture plays an important role, so the monitoring of the crops using the new technology is a useful tool nowadays for the farmer’s job.

**Figure 1:**
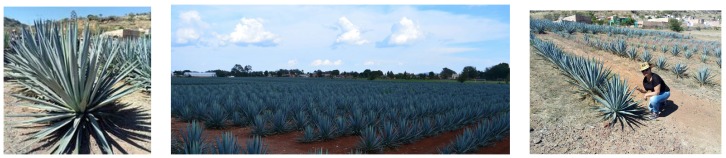
Agave plants and crops.

Agave is one of the important crops of Mexico. Agave is a plant with an American origin, with long leaves in a rosette shape (see Figure 1). Native to the hot and arid regions of Mexico and the Southwestern United States, the *Agave Tequilana Weber* plant is the one used to elaborate tequila, an original Mexican drink [33].

Ref. [34] reports the study of Agave plants through LANDSAT 7 imagery using supervised and unsupervised classification techniques. They used regression and classification tree methods and achieved 70% of Agave accuracy. The authors in Garnica et al. [34] faced the following problems: plant density in agave crops is not homogeneous, agave occurs in many soil types, humidity resulted in a problem for satellite imagery classification, and the agave is camouflaged with other covers like low tropical forest and grasslands, mainly, [34]. To mitigate the influence of all of these problems in the classification process of agave, a possible solution is the use of UAVs, enabled with high resolution cameras.

In this work, we focus on developing a solution for the monitoring of agave crops, taking advantage of the opportunity to obtain a high spatial resolution, which is provided through low-cost UAVs. For the classification, we use an unsupervised approach: *k*-means. During the process, we obtain ortho-mosaics, which allow us to separate plants from other elements in the cultivation. The proposed approach let us perform an inspection of agave crops; in this way, detailed monitoring that helps agave farmers in their daily work can be done. It is worth mentioning that the proposed approach could be used as well for other kinds of plants.

The rest of the paper is organized as follows: Materials and Methods are introduced in Section 2; the proposed Method is presented in Section 3; Section 4 presents the evaluation of the proposed approach, and in the final Section 5, conclusions are given and lines of future work are envisaged.

## 2. Materials and Methods

### 2.1. Work-Flow

**Figure 2:**
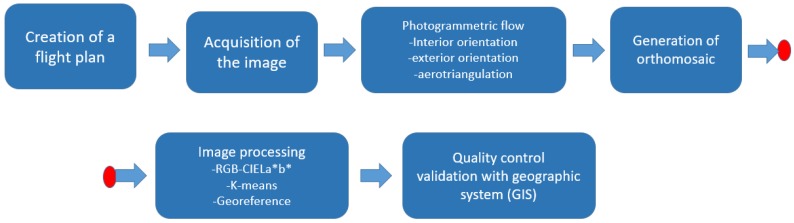
Work-flow system.

In order to describe our research, the photogrammetric process is first presented in general terms, taking into account the state of the art. Then, a new methodology for classifying agave plants is explained. The methodology is based on photogrammetry and a k-means algorithm. Figure 2 illustrates all steps of our methodology.

### 2.2. Study Areas and UAV Flight Plan

**Figure 3:**
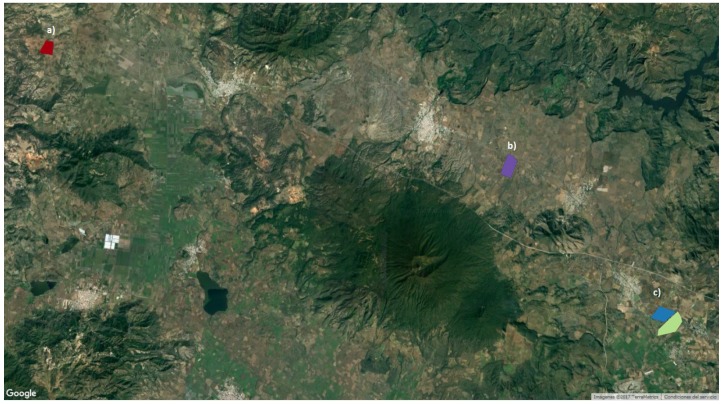
Selected study areas. Regions in red and blue depict areas **a** and **b**, respectively, regions in blue and green correspond to area **c**.

This study looked at four agave areas managed by the Tequila Regulatory Council (CRT). These areas contain information of agave plants of different sizes, concentration and years of age. The area **a**, represented in red in Figure 3, has 3.2 ha approximately and its over flight coordinates are $20^{\circ }$$56^{\prime }$4.76${}^{\prime }$${}^{\prime }$ N, $104^{\circ }$$6^{\prime }$23.70${}^{\prime }$${}^{\prime }$ W. The area **b**, in purple, on the top right-hand side, has 5.8 ha and coordinates of $20^{\circ }$$51^{\prime }$28.24${}^{\prime }$${}^{\prime }$ N, $103^{\circ }$$46^{\prime }$48.92${}^{\prime }$${}^{\prime }$ W . The areas in blue and green are indicated with **c**, on the bottom right of the Figure 3, and they represent around 4.6 ha of land with over flight coordinates $20^{\circ }$$45^{\prime }$14.24${}^{\prime }$${}^{\prime }$ N, $103^{\circ }$$39^{\prime }$57.03${}^{\prime }$${}^{\prime }$ W. The immediate areas, of the previous areas, are included in this study.

The UAV flight plan was checked in advance via Google Earth (Google Inc-DigitalGlobe 2016, Mountain View, CA, USA), the take-off and landing area were specified. The flight time was about 15 min. We performed flight at different altitudes between 40 mts and 100 mts. The flight of the UAV allowed us to obtain the collection of images and their approximate coordinates. It is known that the conditions of the area, weather and the global positioning system generate errors in the image. For this reason, geodetic control points (GCPs) were distributed in the region of interest using the differential GPS.

**Figure 4:**
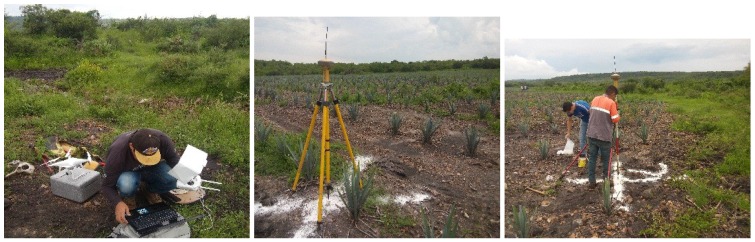
Ground Control Points, (GCPs), generation.

The GCPs provide precision and at least three points are required. However, in our case, we used eight different control points for each studied area, with the aim of minimizing the error in georeferencing [35] (see Figure 4).

### 2.3. Description of the Sensor

**Figure 5:**
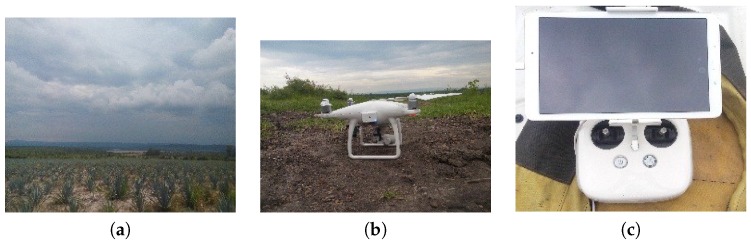
Low cost equipment and field work in selected study areas. (**a**) Agave crop area; (**b**) Unmanned Aerial Vehicle, (UAV); (**c**) Remote Controller.

For the image acquisition, we used a quad-copter Phantom 4 (DJI, Shenzhen, China), see Figure 5. It is low cost equipment, and it has an obstacle detection system of 0.7 to 15 m. Its operating environment must have good illumination to meet the objective. It has Global Positioning System-Global Navigation Satellite System, (GPS-GLONASS) mode, stabilization of 3 axes with a degree of inclination of $-90^{\circ }$ to +30 and axes of horizontal movement, vertical and rotation. The operating distance is about 3 km, and the quad-copter always flies within a clear line of sight for safety reasons. In addition to the automatic flight plan, all of the members of the team also have a manual control of the UAV and therefore the level of skills and knowledge about the manual control should be high, due to the setbacks that can arise directly in the acquisition area.

The quad-copter has a sensor sensitive to Red (R), Green (G), Blue (B) light, (RGB sensor), which allows the capture of image size of 4000 × 3000, from a height predefined by the user. The use of stabilizers allows absorbing the vibration and stabilizes the position of the sensor. The stabilizer is mounted on a gimbal platform that allows for obtaining the searched-for nadir in the images. Table 1 summarizes the main characteristics of our UAV.

### 2.4. Camera Calibration

We used a chessboard pattern approach for camera calibration and we obtained 16 calibration images in different orientations. For this purpose, we used the Camera Calibration Toolbox of Matlab (v. 2012, MathWorks, Inc., Natick, MA, USA) [37,38]. The results of camera calibration process are shown in Table 2.

The calibration parameters allows us to extract the information of the image. The data generated by the calibration process provide a mapping from the image to the real-world dimensions [40].

The parameter that indicates the size of the pixel is called Ground Sample Distance (GSD), and it can be calculated through the Ground Sampling Distance Calculator tool by Pix4D in.

The computation of the size is done according to the following equation:

(1) $$\begin{array}{ccc} GSD & = & \frac{Sw\cdot H\cdot 100}{Fr\cdot imW}, \end{array}$$

where *GSD* is the Ground Sampling Distance (centimeters/pixel) and represents the distance between two consecutive pixel centers, *Sw* denotes the sensor width of the camera (millimeters), *H* is the flight height (meters), *Fr* is the real focal length of the camera (millimeters) and *imW* is the image width (pixels). In our case, Sw=6.25 mm, the average of the flight height was H=60 m, Fr=3.6 mm and the imW=4000 pixels, and, therefore, the distance between the centers of two pixels is 2.6 cm. For H∈[40,80], the *GSD* ∈[1.74,3.47]. The variation in altitude in the previous range did not affect the quality of the agave detection. Therefore, we suggest to use H=60 m, in order to avoid obstacles during the flight and, in some sense, increase the time flight using the same battery.

### 2.5. Photogrammetric Flow

In order to obtain a good result in the image processing, a set of processing steps must be carried out [35]. Currently, in the market, there are a variety of photogrammetric software packages that can perform processes on the UAV images. These packages usually use an algorithm called *structure from motion* that is a set of techniques of photogrammetry and computer vision [41]. In our case, we use the software called Inpho UAS Master 6.0 (Trimble Inc, Sunnyvale, CA, USA) [42] and the application ExifTool that allows to read the metadata of a variety of photographic formats [43].

The starting point for a typical photogrammetric flow are the images set acquired during the flight. In general, all images are georeferenced [44].

In the integration process of photogrammetric flow, the most important phases are:

1. The interior orientation: it refers to the internal geometry of the camera and defines the coordinates of the principal point and focal length.
2. The exterior orientation: [45] It refers to coordinates system projection and attitude (roll, pitch and yaw), which allow for specifying, for each single image, the real position in space. These parameters may be included to Exchangeable Image File Format (EXIF-metadata) [43].
3. The aerial triangulation: it delivers 3D positions of points, measured on images, in a ground control coordinate system. This process consists in generating the correct overlap of each image [46], which, in our case, was in the horizontal of 70% and in the vertical of 30%.

**Figure 6:**
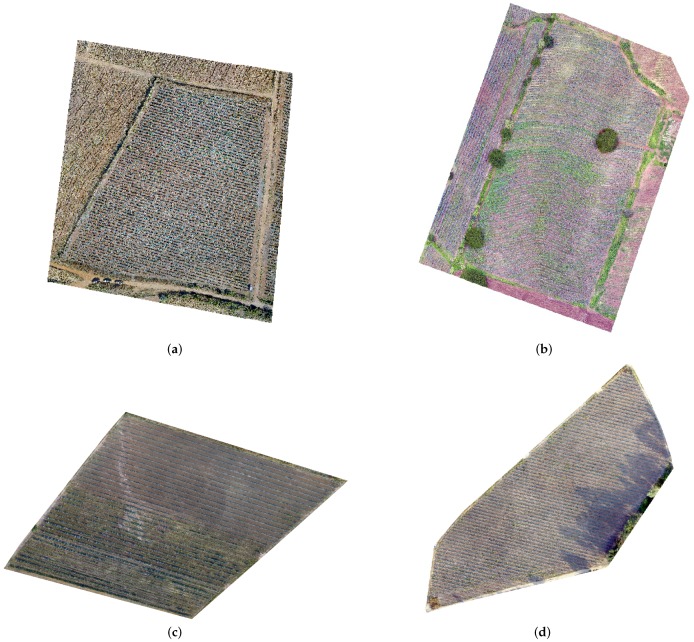
Ortho-mosaics processed with Inpho UAS Master corresponding to: (**a**) area **a**; (**b**) area **b**. Images in panels (**c**,**d**) correspond to polygons in the study area **c**.

We use the Root Mean Square Error (RMSE), to measure the quality of the aerial triangulation. This indicator is based on the residuals of the image coordinates and the ground coordinates. Taking into account conventional aerial photography, an RMSE of up to 1 pixel is desirable; however, according to Laliberte et al. [47], and due to larger distortion of the imagery obtained with low-cost cameras, an acceptable RMSE error is considerable of 1.5 to 2 pixels from the aerial triangulation for UAV imagery (see Table 3). Once aerial triangulation process is finished, a digital terrain model (DTM) can be generated by a dense image matching. The ortho-mosaic can be generated from UAV-based images with known camera parameters and the obtained DTM (see Figure 6). The accuracy values for DTM were: 0.08 m for the area **a**, 0.11 m for the area **b** and 0.07 m for the area **c**. The described procedure is automated by Inpho UAS Master, (Trimble Inc, Sunnyvale, CA,USA) in order to improve the quality of the image.

As a result of this process, we obtain a georeferenced ortho-mosaic image in GeoTIFF file format.

## 3. Image Processing

In our approach, we process the information corresponding to the regions located between 380 nm and 780 nm of the electromagnetic spectrum, i.e., the red (R), green (G) and blue (B) bands. The RGB ortho-mosaic is transformed into the International Commission on Illumination (**C**ommission **I**nternationale de l’**é**clairage), CIE L*a*b* color space. The CIE L*a*b* was developed by the International Commission on Illumination (CIE - Commission International de lÉlairage). CIE color spaces have the capacity to represent perceived color differences across Euclidean distance and are considered as an approximation of the human visual system [48]. For that reason, CIE color spaces are perceptually uniform. In order to convert from the RGB color space to the CIE space L*a*b*, it is first necessary to obtain the so-called artificial primaries, denoted as *X, Y, Z* [48]. The CIE *XYZ* space is the result of direct measurements on the human eye made in the late 1920s by W. David Wright [49] and John Guild [50] and serves as the basis for other color representations. The values of *XYZ* are calculated by means of linear transformation of the RGB given by the Expression (2):

(2) $$\left[\begin{array}{c} X\\ Y\\ Z \end{array}\right]=\left[\begin{array}{ccc} 0.4125 & 0.3576 & 0.1804\\ 0.2125 & 0.7154 & 0.0721\\ 0.0193 & 0.1192 & 0.9502 \end{array}\right]\left[\begin{array}{c} R\\ G\\ B \end{array}\right].$$

In (2), the values of R, G and B are in the interval [0,1]. The elements of the transformation matrix depend on the type of selected reference white [48,51], and these values are tabulated in [48]. We considered D65 reference white [48], which is usually used for standard RGB monitors (sRGB) [52]. The values in the space L*a*b* are calculated from the *XYZ*, by a non-linear transformation, see Equations (3)–(5):

(3) $$L^{*}=116\left(\sqrt[{3}]{\frac{Y}{Y_{0}}}\right)-16,$$

(4) $$a^{*}=500\left(\sqrt[{3}]{\frac{X}{X_{0}}}-\sqrt[{3}]{\frac{Y}{Y_{0}}}\right),$$

(5) $$b^{*}=200\left(\sqrt[{3}]{\frac{Y}{Y_{0}}}-\sqrt[{3}]{\frac{Z}{Z_{0}}}\right).$$

In Equations (3)–(5) $X_{0}$, $Y_{0}$ and $Z_{0}$ are the values corresponding to the RGB vector [1,1,1], i.e., the white color in RGB color space. For details of the implementation, see the information described in http://www.brucelindbloom.com/.

**Figure 7:**
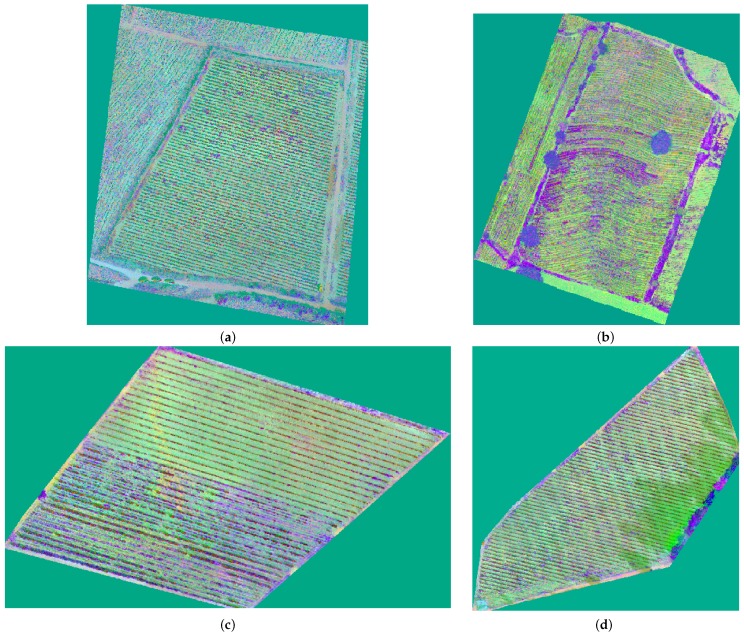
Ortho-mosaics represented in CIE L*a*b color space: (**a**) Ortho-mosaic corresponding to area **a**; (**b**) Ortho-mosaic corresponding to area **b**. Images in panels (**c**,**d**) correspond to polygons in the study area **c**.

In Figure 7, the examples of the results are shown corresponding to the color space CIE L*a*b*.

After the color space transformation, the k-means algorithm is applied on the CIE L*a*b* ortho-mosaic. This approach is a non-supervised learning algorithm, which allows for generating different class groups. k-means uses the distance criterion as a measure of similarity, and it is widely used in scientific classification schemes and in the field of pattern recognition [53]. The criterion distance justifies the use of the CIE L*a*b*.

According to the research in [34], an unsupervised approach is a feasible strategy for agave monitoring. Supervised algorithms require good samples and enough samples for the training step, and, in the case of agave study, it is very difficult to have training samples without other land covers. This being the reason, in our proposal, we use an unsupervised algorithm in order to separate the plantations of agave in relation to other land covers.

The k-means algorithm [54] allows us to create two segmented layers: agave plant and weeds.

Some authors addressed the computational limitations of k-means [55]. In order to improve the performance of the k-means, we use a parallel approach [56].

**Figure 8:**
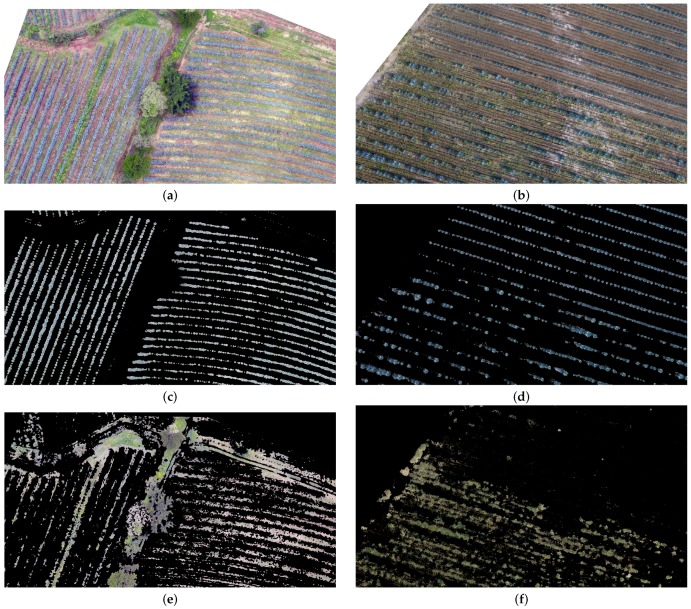
Example of classification results by *k*-means. (**a**) Image sample of the study area **b**; (**b**) Image sample of the study area **c**; (**c**) Detected agave plants in the sample represented in panel (**a**); (**d**) Detected agave plants in the image represented in panel (**b**); (**e**) Detected weeds in the sample represented in panel (**a**); (**f**) Detected weeds in the image represented in panel (**b**).

In Figure 8, an example of the results of classification through k-means is depicted. The image represented in Figure 8 a,b corresponds to regions located in study areas **b** and **c**, indicated in Figure 3.

After the classification step through *k*-means, we create a copy of the geographic data of our ortho-mosaic. The geographic data are extracted from the GeoTIFF file [57,58]. The created copy is annexed to the file created by means of *k*-means. We used the Matlab (v.2012) implementation of *k*-means and GeoTIFF procedures. The programs that we elaborated in Matlab allows users to fix all necessary parameters. We carried out several experiments in order to find the best number of classes, *k*, for the *k*-means algorithm. According to our results, k=3 was the best value of *k*, because, with this value, the agave plants and weeds were best discriminated. The third group detected regions not relevant for our application. In our study, we use the computer workstation with a high performance processors: Intel^®^ Xeon^®^ (Intel Corporation, Santa Clara, CA, USA) E3-1280 v5 3.7 GHz, up to 4 GHz with Intel Turbo Boost Technology, 8 MB cache, 4 cores, with Ram memory 32 GB DDR4 and with a Serial ATA, hard drive (SATA technology, Beaverton, OR, USA) with 2 TB storage [55].

## 4. Evaluation of Methodology

The accuracy of the processing in the described methodology depends mainly on three aspects: on the resolution of the UAV sensor, on the photogrammetric process and on the georeference. In order to evaluate our proposal, 25 samples were taken at different sites around the area of interest: 10 of them represent weeds and 15 agave plants. Each sample was obtained with relative accuracy planimetric [59] through the georeferenced ortho-mosaic, comparing this image with segmented images of agave plants and weed on a geographic information system QGIS (Quantum Geographic Information System v2.162, Project of the Open Source Geospatial Foundation, Beaverton, OR, USA). With this procedure, we gather the information about the position of plants or weed areas into conformance with the Universal Transverse Mercator (UTM) map projection [60].

**Figure 9:**
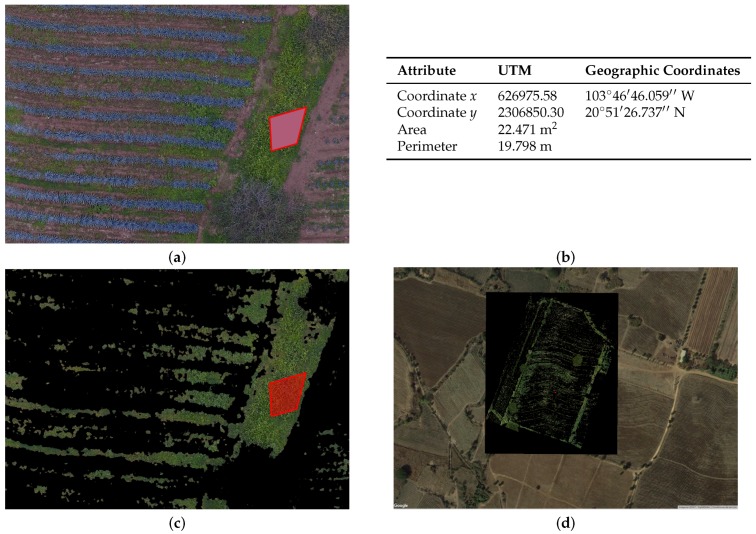
(**a**) example of sample weed, showed in the QGIS system (polygon in red), (**b**) table with the object attributes described by QGIS, (**c**) segmentation of the weed sample by *k*-means (polygon in red), (**d**) full study area **b**.

**Figure 10:**
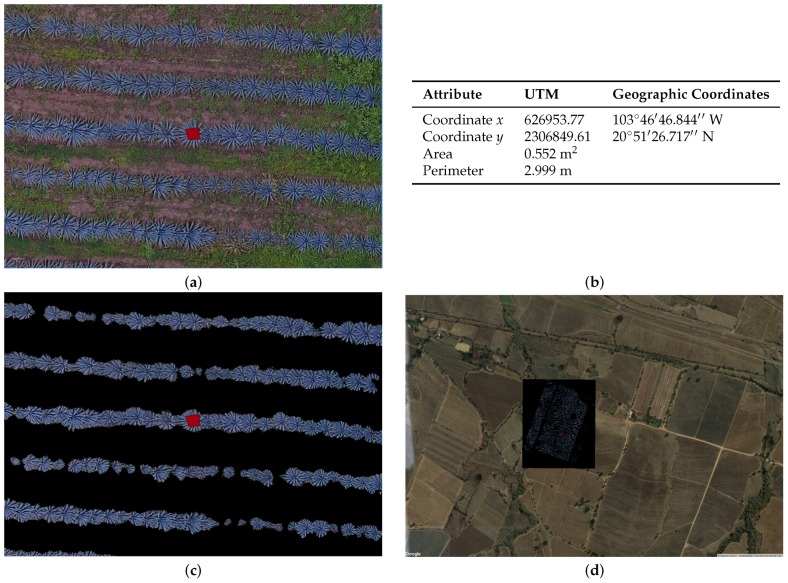
(**a**) position of agave plants in the QGIS system; (**b**) table with the object attributes described by QGIS; (**c**) sample of detected plants; and (**d**) full study area **b** described in Section 2.2, plants’ segmentation, shown in Google earth.

Figure 9 and Figure 10 depict how we can validate the results obtained from the segmentation of agave plants and weed.

First, we create random polygons of agave and weed. Then, we apply the identify tool in QGIS on every single polygon, and we verify the information output and the attributes for both agave and weed (see tables in Figure 9b and Figure 10b).

After checking the spatial information, the segmentation results of the selected area are verified in the field by the Tequila Regulatory Council (CRT) in Mexico.

It is worth mentioning that this process has been applied to all of the acquired images. In total, we processed four ortho-mosaics, and, for all of them, we obtained a valuable result. Table 4 contains the numerical evaluation of the segmentation of the agave plants by k-means. As it can be seen, obtained results are all over 99.999% in accuracy when compared to the human made process, which has been considered by the Agave Regulation Agency as a very good result. Study areas in column 1 correspond to the areas described in Section 2.2.

**Figure 11:**
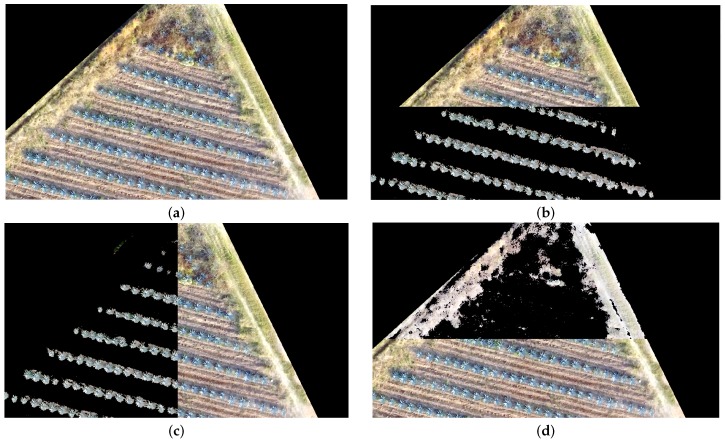
Detection of agave plants and weed corresponding to the study area **c** Described in Section 2.2: (**a**) Original ortho-mosaic; (**b**) Agave detection bottom–top; (**c**) Agave detection left–right; and (**d**) Detected weed.

**Figure 12:**
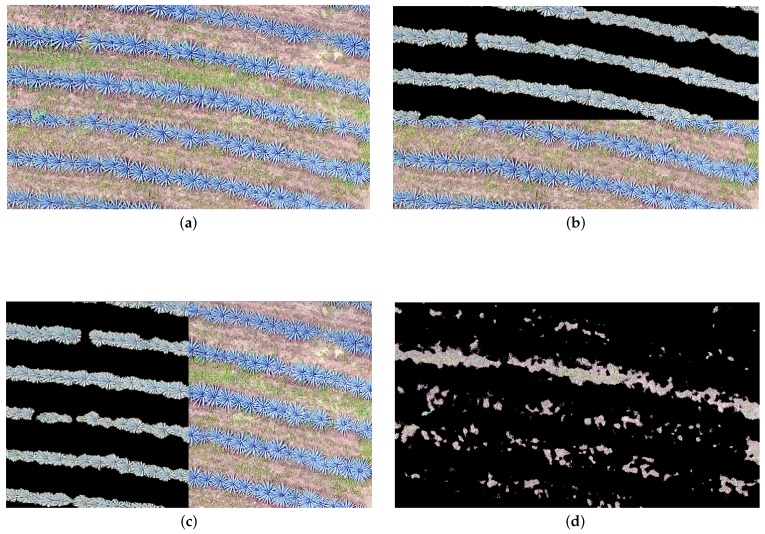
Detection of agave plants and Weed corresponding to the study area **b** Described in Section 2.2: (**a**) Original ortho-mosaic; (**b**) Agave detection top–bottom; (**c**) Agave detection left–right; and (**d**) Detected weed.

Figure 11 and Figure 12 illustrate an example of the segmentation results. In both figures, (a) represents the studied land part, and (b), (c) and (d) represent the overlap between the original and segmented images.

## 5. Conclusions

In this work, we proposed a methodology for agave crop monitoring. The methodology combines remote sensing through low-cost UAV, photogrammetry, computer vision, data mining, geomatics and computer science. This study has demonstrated the potential development of low-cost unmanned aerial vehicles in the area of agave monitoring. We achieved excellent detection results, which is demonstrated by the obtained precision value of 99%. The monitoring of the vegetation through UAV will allow, in the near future, the generation of very important data for the study of plants such as agave. The results of this study is the base for the geospatial database, which we are building to analyze the behavior of the agave plants. At the moment, we work together with the Tequila Regulatory Council in Mexico. To the best of our knowledge, this is the first application that integrates remote sensing based on low cost UAV, image processing and pattern recognition techniques for georeferenced images for agave crop monitoring.

As future work, an extension of the presented approach is envisaged, in order to apply it to wider areas of agave and help farmers in other places different to those used in the experimental phase. The presented approach could be applied as well to supervise other types of plants; an improvement of the model is needed to this end, in order to adapt to the characteristics of the plant of interest.

## Figures and Tables

**Table 1:** General characteristics of Unmanned Aerial Vehicle (UAV) [36].

Parameter	Value	
Sensor RGB	6.25 mm × 4.68 mm
Weight	25 grams
Sensor	12.4 Megapixels
Lens	FOV $94^{\circ }$
Focal length	20 mm (35 mm format equivalent) f/2.8 focus at *∞*
Pixel size	1.5625 μm
Measurement of image	4000 × 3000
Image Type	JPEG, DNG (RAW)
Temperature	$0^{\circ }$ to $40^{\circ }$

**Table 2:** Camera calibration parameter values [39].

Parameter	Values
Focal length	(2.2495 $\times 10^{3},$ 2.2498 $\times 10^{3}$)
Principal point coordinates	(2.0159 $\times 10^{3}$ , 1.5088 $\times 10^{3}$)
Skew	−7.2265
Lens distortion	
Tangential Distortion coefficients	(0.0011, 5.6749 $\times 10^{-4}$)
Radial distortion coefficients	(−0.0160, −0.0336)
Num. Patterns	16

**Table 3:** Root Mean Square Error (RMSE) aerial triangulation.

Study Areas	Images Low-Cost Camera	Number of Flight Lines	Image Resolution (cm)	RMSE (Pixels/cm)
**a**	146	10	2.60	1.4
**b**	140	8	1.63	1.7
**c**	266	18	2.10	1.6

**Table 4:** Plant detection accuracy.

Study Areas	Precision	Overall Accuracy
**a**	0.99995	0.99994
**b**	0.99998	0.99998
**c**	0.99961	0.99961
**d**	0.99991	0.99998
